# Comparison between proximal thoracic vascular measurements obtained by contrast enhanced MRA and transthoracic echocardiography in infants with congenital heart disease

**DOI:** 10.1186/1532-429X-14-S1-P121

**Published:** 2012-02-01

**Authors:** Nitin Madan, Jen Yau, Shubhika Srivastava, James Nielsen

**Affiliations:** 1 Pediatrics, Mount Sinai School of Medicine, New York, NY, USA

## Background

Accurate assessment of the proximal thoracic vasculature in infants with congenital heart disease (CHD) is vital in deciding the appropriate surgical or interventional procedure and predicting outcomes. In most infants this information is obtained by transthoracic echocardiography (TTE). When the image quality is limited by TTE, Contrast Enhanced MRA (CE-MRA) is frequently utilized. Calculating Z-scores for measurements obtained by CE-MRA in this population is currently not possible due to the lack of normative data. Demonstrating reasonable agreement between vessel dimensions by CE-MRA and TTE will allow the use of TTE based Z-scores on measurements from CE-MRA. This study examines the accuracy and agreement of proximal thoracic vascular measurements obtained by CE-MRA compared to TTE.

## Methods

Infants with CHD who had a TTE study in close proximity to a CE-MRA between August 2006 and May 2011 were identified. Main pulmonary artery, branch pulmonary arteries, ascending aorta, distal transverse arch and aortic isthmus were measured from CE-MRA and TTE in analogous imaging planes and locations by two investigators blinded to each other. TTE measurements were performed at end-systole.

## Results

**Figure 1:**
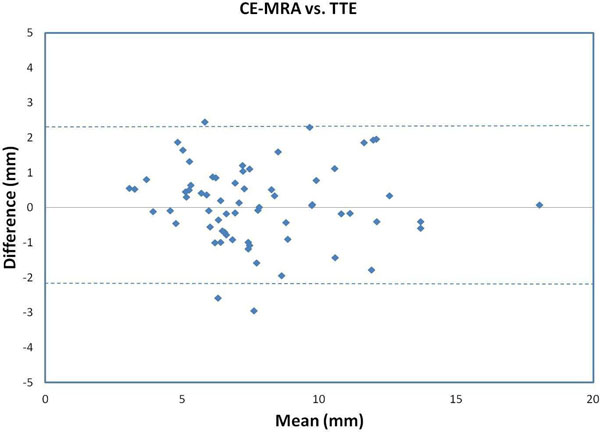
Bland Altman plot for CE-MRA vs. TTE. Dash line equals limits of agreement.

18 subjects with CHD, median age 79 days (1 to 238 days), weight 4.1 kg (2.16-9.3 kg), who had a CE-MRA and TTE were included in the study. Data was analyzed from 67 paired measurements after excluding 41 measurements due to inadequate visualization of vessel borders. The time period between the two imaging modalities was a median of 5 days (0-60 days). The range of vessel size was between 2.8 mm to 18 mm. The correlation between CE-MRA and TTE was excellent (r=0.93, p<0.001). The mean difference between the measurements was 0.08 mm ± 1.1 mm with limits of agreement of -2.1 mm to 2.3 mm (Figure 1).

## Conclusions

The agreement between CE-MRA and TTE measurements in infants from this cohort is favorable and would support using echocardiographic Z-scores for thoracic vessels on CE-MRA data.

## Funding

None.

